# A Non-Linear Filtering Algorithm Based on Alpha-Divergence Minimization

**DOI:** 10.3390/s18103217

**Published:** 2018-09-24

**Authors:** Yarong Luo, Chi Guo, Jiansheng Zheng, Shengyong You

**Affiliations:** Global Navigation Satellite System Research Center, Wuhan University, Wuhan 430079, China; yarongluo@whu.edu.cn (Y.L.); zjs@whu.edu.cn (J.Z.); shengyongyou@whu.edu.cn (S.Y.)

**Keywords:** alpha-divergence, Kullback–Leibler divergence, non-linear filtering, exponential family distribution

## Abstract

A non-linear filtering algorithm based on the alpha-divergence is proposed, which uses the exponential family distribution to approximate the actual state distribution and the alpha-divergence to measure the approximation degree between the two distributions; thus, it provides more choices for similarity measurement by adjusting the value of α during the updating process of the equation of state and the measurement equation in the non-linear dynamic systems. Firstly, an α-mixed probability density function that satisfies the normalization condition is defined, and the properties of the mean and variance are analyzed when the probability density functions p(x) and q(x) are one-dimensional normal distributions. Secondly, the sufficient condition of the alpha-divergence taking the minimum value is proven, that is when α≥1, the natural statistical vector’s expectations of the exponential family distribution are equal to the natural statistical vector’s expectations of the α-mixed probability state density function. Finally, the conclusion is applied to non-linear filtering, and the non-linear filtering algorithm based on alpha-divergence minimization is proposed, providing more non-linear processing strategies for non-linear filtering. Furthermore, the algorithm’s validity is verified by the experimental results, and a better filtering effect is achieved for non-linear filtering by adjusting the value of α.

## 1. Introduction

The analysis and design of non-linear filtering algorithms are of enormous significance because non-linear dynamic stochastic systems have been widely used in practical systems, such as navigation system [1], simultaneous localization and mapping [2], and so on. Because the state model and the measurement model are non-linear and the state variables and the observation variables of the systems no longer satisfy the Gaussian distribution, the representation of the probability density distribution of the non-linear function will become difficult. In order to solve this problem, deterministic sampling (such as the unscented Kalman filter and cubature Kalman filter) and random sampling (such as the particle filter) are adopted to approximate the probability density distribution of the non-linear function, that is to say, to replace the actual state distribution density function by a hypothetical one [3].

In order to measure the similarity between the hypothetical state distribution density function and the actual one, we need to select a measurement method to ensure the effectiveness of the above methods. The alpha-divergence, proposed by S.Amari, is used to measure the deviation between data distributions p(x) and q(x) [4]. It can be used to measure the similarity between the hypothetical state distribution density function and the actual one for the non-linear filtering. Compared with the Kullback–Leibler divergence (the KL divergence), the alpha-divergence provides more choices for measuring the similarity between the hypothetical state distribution density function and the actual one. Therefore, we use alpha-divergence as a measurement criterion to measure the similarity between the two distribution functions. Indeed, adjusting the value of parameter α in the function can ensure the interesting properties of similarity measurement. Another choice of α characterizes different learning principles, in the sense that the model distribution is more inclusive (α→∞) or more exclusive (α→−∞) [5]. Such flexibility enables α-based methods to outperform KL-based methods with the value of α being properly selected. The higher the similarity of the two probability distributions p(x) and q(x), the smaller the value of alpha-divergence will be. Then, it can be proven that in a specific range of value, q(x) can fully represent the properties of p(x) when the value of alpha-divergence is minimum.

Because the posterior distribution of non-linear filtering is difficult to solve, given that the posterior probability distribution is p(x), we can use the probability distribution q(x) to approximate the posterior probability distribution p(x) of non-linear filtering. The approximate distribution q(x) is expected to be a distribution with a finite moment vector. This in turn means that a good choice for the approximate distribution is from the exponential family distribution, which is a practically convenient and widely-used unified family of distributions on finite dimensional Euclidean spaces.

The main contributions of this article include:

1. We define an α-mixed probability density function and prove that it satisfies the normalization condition when we specify the probability distributions p(x) and q(x) to be univariate normal distributions. Then, we analyze the monotonicity of the mean and the variance of the α-mixed probability density function with respect to the parameter when p(x) and q(x) are specified to be univariate normal distributions. The results will be used in the algorithm implementation to guarantee the convergence.
2. We specify the probability density function q(x) as an exponential family state density function and choose it to approximate the known state probability density function p(x). After the α-mixed probability density function is defined by q(x) and p(x), we prove that the sufficient condition for alpha-divergence minimization is when α≥1 and the expected value of the natural statistical vector of q(x) is equivalent to the expected value of the natural statistical vector of the α-mixed probability density function.
3. We apply the sufficient condition to the non-linear measurement update step of the non-linear filtering. The experiments show that the proposed method can achieve better performance by using a proper α value.

## 2. Related Work

It has become a common method to apply various measurement methods of divergence to optimization and filtering, among which the KL divergence, as the only invariant flat divergence, has been most commonly studied [6]. The KL divergence is used to measure the error in the Gaussian approximation process, and it is applied in the process of distributing updated Kalman filtering [7]. The proposal distribution of the particle filter algorithm is regenerated using the KL divergence after containing the latest measurement values, so the new proposal distribution approaches the actual posterior distribution [8]. Martin et al. proposed the Kullback–Leibler divergence-based differential evolution Markov chain filter for global localization for mobile robots in a challenging environment [9], where the KL-divergence is the basis of the cost function for minimization. The work in [3] provides a better measurement method for estimating the posterior distribution to apply KL minimization to the prediction and updating of the filtering algorithm, but it only provides the proof of the KL divergence minimization. The similarity of the posterior probability distribution between adjacent sensors in the distributed cubature Kalman filter is measured by minimizing the KL divergence, and great simulation results are achieved in the collaborative space target tracking task [10].

As a special situation of alpha-divergence, the KL divergence is easy to calculate, but it provides only one measurement method. Therefore, the studies on the theory and related applications of the KL divergence are taken seriously. A discrete probability distribution of minimum Chi-square divergence is established [11]. Chi-square divergence is taken as a new criterion for image thresholding segmentation, obtaining better image segmentation results than that from the KL divergence [12,13]. It has been proven that the alpha-divergence minimization is equivalent to the α-integration of stochastic models, and it is applied to the multiple-expert decision-making system [6]. Amari et al. [14] also proved that the alpha-divergence is the only divergence category, which belongs to both f-divergence and Bregman divergence, so it has information monotonicity, a geometric structure with Fisher’s measurement and a dual flat geometric structure. Gultekin et al. [15] proposed to use Monte Carlo integration to optimize the minimization equation of alpha-divergence, but this does not prove the alpha-divergence minimization. In [16], the application of the alpha-divergence minimization in approximate reasoning has been systematically analyzed, and different values of α can change the algorithm between the variational Bayesian algorithm and expectation propagation algorithm. As a special situation of the alpha-divergence (α=2q−1), *q*-entropy [17,18] has been widely used in the field of physics. Li et al. [19] proposed a new class of variational inference methods using a variant of the alpha-divergence, which is called Rényi divergence, and applied it to the variational auto-encoders and Bayesian neural networks. There are more introductions about theories and applications of the alpha-divergence in [20,21]. Although the theories and applications of alpha-divergence have been very popular, we focus on providing a theory to perfect the alpha-divergence minimization and apply it to non-linear filtering.

## 3. Background Work

In Section 3.1, we provide the framework of the non-linear filtering. Then, we introduce the alpha-divergence in Section 3.2, which contains many types of divergence as special cases.

### 3.1. Non-Linear Filtering

The actual system studied in the filtering is usually non-linear and non-Gaussian. Non-linear filtering refers to a filtering that can estimate the optimal estimation problem of the state variables in the dynamic system online and in real time from the system observations.

The state space model of non-linear systems with additive Gaussian white noise is:

(1) $$x_{k}=f\left(x_{k-1}\right)+w_{k-1}$$

where $x_{k}\in R^{n}$ is the system state vector that needs to be estimated; $w_{k}$ is the zero mean value Gaussian white noise, and its variance is $E\left[w_{k}w_{k}^{T}\right]=Q_{k}$. Equation (1) describes the state transition $p(x_{k}|x_{k-1})$ of the system.

The random observation model of the state vector is:

(2) $$z_{k}=h\left(x_{k}\right)+v_{k}$$

where $z_{k}\in R^{m}$ is system measurement; $v_{k}$ is the zero mean value Gaussian white noise, and its variance is $E\left[v_{k}v_{k}^{T}\right]=R_{k}$. Suppose $w_{k}$ and $v_{k}$ are independent of each other and the observed value $z_{k}$ is independent of the state variables $x_{k}$.

**Figure 1:**
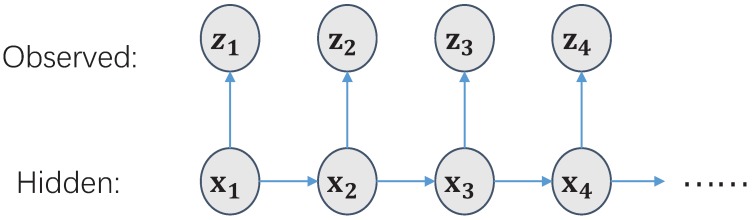
Hidden Markov Model (HMM).

The entire probability state space is represented by the generation model as shown in Figure 1. $x_{k}$ is the system state; $z_{k}$ is the observational variable, and the purpose is to estimate the value of state $x_{k}$. The Bayesian filter is a general method to solve state estimation. The Bayesian filter is used to calculate the posterior distribution $p(x_{k}|z_{k})$, and its recursive solution consists of prediction steps and update steps.

Under the Bayesian optimal filter framework, the system state equation determines that the conditional transition probability of the current state is a Gaussian distribution:

(3) $$p\left(x_{k}|x_{k-1},z_{1:k-1}\right)=N\left(x_{k}|f\left(x_{k-1}\right),Q_{k}\right)$$

If the prediction distribution of the system can be obtained from Chapman–Kolmogorov, the prior probability is:

(4) $$p\left(x_{k}|z_{1:k-1}\right)=\int p\left(x_{k}|x_{k-1},z_{1:k-1}\right)p\left(x_{k-1}|z_{1:k-1}\right)dx_{k-1}$$

When there is a measurement input, the system measurement update equation determines that the measurement likelihood transfer probability of the current state obeys a Gaussian distribution:

(5) $$p\left(z_{k}|x_{k},z_{1:k-1}\right)=N\left(z_{k}|h\left(x_{k}\right),R_{k}\right)$$

According to the Bayesian information criterion, the posterior probability obtained is:

(6) $$p\left(x_{k}|z_{1:k}\right)=\frac{p\left(z_{k}|x_{k},z_{1:k-1}\right)p\left(x_{k}|z_{1:k-1}\right)}{p(z_{k}|z_{1:k-1})}$$

where $p(z_{k}|z_{1:k-1})$ is the normalized factor, and it is defined as follows:

(7) $$p\left(z_{k}|z_{1:k-1}\right)=\int p\left(z_{k}|x_{k},z_{1:k-1}\right)p\left(x_{k}|z_{1:k-1}\right)dx_{k}$$

Unlike the Kalman filter framework, the Bayesian filter framework does not demand that the update structure be linear, so it can use non-linear update steps.

In the non-linear filtering problem, the posterior distribution $p(x_{k}|z_{1:k})$ often cannot be solved correctly. Our purpose is to use the distribution q(x) to approximate the posterior distribution $p(x_{k}|z_{1:k})$ without an analytical solution. Here, we use the alpha-divergence measurement to measure the similarity between the two. We propose a method that directly minimizes alpha-divergence without adding any additional approximations.

### 3.2. The Alpha-Divergence

The KL divergence is commonly used in similarity measures, but we will generalize it to the alpha-divergence. The alpha-divergence is a parametric family of divergence functions, including several well-known divergence measures as special cases, and it gives us more flexibility in approximation [20].

**Definition** **1.** *Let us consider two unnormalized distributions p(x) and q(x) with respect to a random variable x. The alpha-divergence is defined by:*

(8) $$D_{\alpha }\left[p\|q\right]=\frac{1}{\alpha (1-\alpha )}\int \alpha p\left(x\right)+\left(1-\alpha \right)q\left(x\right)-p{\left(x\right)}^{\alpha }q{\left(x\right)}^{1-\alpha }dx$$

*where α∈R, which means $D_{\alpha }$ is continuous at zero and one.*

The alpha-divergence meets the following two properties:

1. $D_{\alpha }\left[p\|q\right]\geq 0$, if and only if p=q, $D_{\alpha }\left[p\|q\right]=0$. This property can be used precisely to measure the difference between the two distributions.
2. $D_{\alpha }\left[p\|q\right]$ is a convex function with respect to p(x) and q(x).

Note that the term ∫[αp(x)+(1−α)q(x)]dx disappears when p(x) and q(x) are normalized distributions, i.e., ∫p(x)dx=∫p(x)dx=1. The alpha-divergence in (8) is expressed by:

(9) $$D_{\alpha }\left[p\|q\right]=\frac{1}{\alpha (1-\alpha )}\left(1-\int p{\left(x\right)}^{\alpha }q{\left(x\right)}^{1-\alpha }dx\right)$$

In general, we can get another equivalent expression of the alpha-divergence when we set β=2α−1:

(10) $$D_{\beta }\left[p\|q\right]=\frac{4}{1-\beta^{2}}\int \frac{1-\beta }{2}p\left(x\right)+\frac{1+\beta }{2}q\left(x\right)-p{\left(x\right)}^{\frac{1+\beta }{2}}q{\left(x\right)}^{\frac{1-\beta }{2}}dx$$

Alpha-divergence includes several special cases such as the KL divergence, the Hellinger divergence and $\chi^{2}$ divergence (Pearson’s distance), which are summarized below.

- As α approaches one, Equation (8) is the limitation form of $\frac{0}{0}$, and it specializes to the KL divergence from q(x) to p(x) as L’Hôpital’s rule is used:
  (11) $$\begin{array}{cc} \lim_{\alpha \to 1}D_{\alpha }\left[p\|q\right] & =\lim_{\alpha \to 1}\frac{1}{\alpha (1-\alpha )}\int \alpha p\left(x\right)+\left(1-\alpha \right)q\left(x\right)-p{\left(x\right)}^{\alpha }q{\left(x\right)}^{1-\alpha }dx\\ & =\lim_{\alpha \to 1}\frac{1}{1-2\alpha }\int p\left(x\right)-q\left(x\right)-p{\left(x\right)}^{\alpha }log\left(p\left(x\right)\right)q{\left(x\right)}^{1-\alpha }+p{\left(x\right)}^{\alpha }q{\left(x\right)}^{1-\alpha }log\left(q\left(x\right)\right)dx\\ & =\int p\left(x\right)log\frac{p(x)}{q(x)}-p\left(x\right)+q\left(x\right)dx=KL\left[p\|q\right] \end{array}$$
  When p(x) and q(x) are normalized distributions, the KL divergence is expressed as:
  (12) $$KL\left[p\|q\right]=\int p\left(x\right)log\frac{p(x)}{q(x)}dx$$
- As α approaches zero, Equation (8) is still the limitation form of $\frac{0}{0}$, and it specializes to the dual form of the KL divergence from q(x) to p(x) as L’Hôpital’s rule is used:
  (13) $$\begin{array}{cc} \lim_{\alpha \to 0}D_{\alpha }\left[p\|q\right] & =\lim_{\alpha \to 0}\frac{1}{\alpha (1-\alpha )}\int \alpha p\left(x\right)+\left(1-\alpha \right)q\left(x\right)-p{\left(x\right)}^{\alpha }q{\left(x\right)}^{1-\alpha }dx\\ & =\lim_{\alpha \to 0}\frac{1}{1-2\alpha }\int p\left(x\right)-q\left(x\right)-p{\left(x\right)}^{\alpha }log\left(p\left(x\right)\right)q{\left(x\right)}^{1-\alpha }+p{\left(x\right)}^{\alpha }q{\left(x\right)}^{1-\alpha }log\left(q\left(x\right)\right)dx\\ & =\int q\left(x\right)log\frac{q(x)}{p(x)}+p\left(x\right)-q\left(x\right)dx=KL\left[q\|p\right] \end{array}$$
  When p(x) and q(x) are normalized distributions, the dual form of the KL divergence is expressed as:
  (14) $$KL\left[q\|p\right]=\int q\left(x\right)log\frac{q(x)}{p(x)}dx$$
- When $\alpha =\frac{1}{2}$, the alpha-divergence specializes to the Hellinger divergence, which is the only dual divergence in the alpha-divergence:
  (15) $$D_{\frac{1}{2}}\left[p\|q\right]=2\int \left(p\left(x\right)+q\left(x\right)-2p{\left(x\right)}^{\frac{1}{2}}q{\left(x\right)}^{\frac{1}{2}}\right)dx=2\int {\left(\sqrt{p(x)}-\sqrt{q(x)}\right)}^{2}dx=4Hel^{2}\left[p\|q\right]$$
  where $Hel\left[p\|q\right]=\frac{1}{2}\int {\left(\sqrt{p(x)}-\sqrt{q(x)}\right)}^{2}dx$ is the Hellinger distance, which is the half of the Euclidean distance between two random distributions after taking the difference of the square root, and it corresponds to the fundamental property of distance measurement and is a valid distance metric.
- When α=2, the alpha-divergence degrades to $\chi^{2}$-divergence:
  (16) $$\begin{array}{cc} D_{2}\left[p\|q\right] & =\frac{-1}{2}\left(\int 2p\left(x\right)-q\left(x\right)-\frac{p{\left(x\right)}^{2}}{q(x)}dx\right)\\ & =\frac{1}{2}\left(\int \frac{p{\left(x\right)}^{2}+q{\left(x\right)}^{2}-2p\left(x\right)q\left(x\right)}{q(x)}dx\right)=\frac{1}{2}\int \frac{{\left(p\left(x\right)-q\left(x\right)\right)}^{2}}{q(x)}dx \end{array}$$

In the later experiment, we will adapt the value of α to optimize the distribution similarity measurement.

## 4. Non-Linear Filtering Based on the Alpha-Divergence

We first define an α-mixed probability density function, which will be used in the non-linear filtering based on the alpha-divergence minimization. Then, we show that the sufficient condition for the alpha-divergence minimization is when α≥1 and the expected value of the natural statistical vector of q(x) is equivalent to the expected value of the natural statistical vector of the α-mixed probability density function. At last, we apply the sufficient condition to the non-linear measurement update steps for solving the non-linear filtering problem.

### 4.1. The α-Mixed Probability Density Function

We first give a definition of a normalized probability density function called the α-mixed probability density function, which is expressed as $p_{\alpha }\left(x\right)$.

**Definition** **2.** *We define an α-mixed probability density function:*

(17) $$p_{\alpha }\left(x\right)=\frac{p{\left(x\right)}^{\alpha }q{\left(x\right)}^{\left(1-\alpha \right)}}{\int p{\left(x\right)}^{\alpha }q{\left(x\right)}^{\left(1-\alpha \right)}dx}$$

We can prove that when both p(x) and q(x) are univariate normal distributions, then $p_{\alpha }\left(x\right)$ is still the Gaussian probability density function.

Suppose that $p\left(x\right)∼N\left(\mu_{p},\sigma_{p}^{2}\right)$ and $q\left(x\right)∼N\left(\mu_{q},\sigma_{q}^{2}\right)$, so the probability density functions can be expressed as follows:

(18) $$p\left(x\right)=\frac{1}{\sqrt{2\pi }\sigma_{p}}exp\left\{-\frac{{\left(x-\mu_{p}\right)}^{2}}{2\sigma_{p}^{2}}\right\}\;\mathrm{and}\;q\left(x\right)=\frac{1}{\sqrt{2\pi }\sigma_{q}}exp\left\{-\frac{{\left(x-\mu_{q}\right)}^{2}}{2\sigma_{q}^{2}}\right\}$$

Then we can combine these two functions with parameter α:

(19) $$\begin{array}{cc} p{\left(x\right)}^{\alpha }q{\left(x\right)}^{\left(1-\alpha \right)} & ={\left(2\pi \sigma_{p}^{2}\right)}^{-\frac{\alpha }{2}}{\left(2\pi \sigma_{q}^{2}\right)}^{-\frac{1-\alpha }{2}}exp\left\{-\frac{\alpha {\left(x-\mu_{p}\right)}^{2}\sigma_{q}^{2}+\left(1-\alpha \right){\left(x-\mu_{q}\right)}^{2}\sigma_{p}^{2}}{2\sigma_{p}^{2}\sigma_{q}^{2}}\right\}\\ & =\frac{S_{\alpha }}{\sqrt{2\pi }\sigma_{\alpha }}exp\left\{-\frac{{\left(x-\mu_{\alpha }\right)}^{2}}{2\sigma_{\alpha }^{2}}\right\} \end{array}$$

where $\mu_{\alpha }=\frac{\alpha \mu_{p}\sigma_{q}^{2}+\left(1-\alpha \right)\mu_{q}\sigma_{p}^{2}}{\alpha \sigma_{q}^{2}+\left(1-\alpha \right)\sigma_{p}^{2}}$ is the mean of the α-mixed probability density function; $\sigma_{\alpha }^{2}=\frac{\sigma_{q}^{2}\sigma_{p}^{2}}{\alpha \sigma_{q}^{2}+\left(1-\alpha \right)\sigma_{p}^{2}}$ (which can be reduced to $\frac{1}{\sigma_{\alpha }}=\alpha \frac{1}{\sigma_{p}}+\left(1-\alpha \right)\frac{1}{\sigma_{q}}$) is the variance of the α-mixed probability density function; $S_{\alpha }$ is a scalar factor, and the expression is as follows:

(20) $$\begin{array}{cc} S_{\alpha } & ={\left(2\pi \sigma_{\alpha }^{2}\right)}^{\frac{1}{2}}{\left(2\pi \sigma_{p}^{2}\right)}^{-\frac{\alpha }{2}}{\left(2\pi \sigma_{q}^{2}\right)}^{-\frac{1-\alpha }{2}}exp\left\{-\frac{\alpha \left(1-\alpha \right){\left(\mu_{p}-\mu_{q}\right)}^{2}}{2[\alpha \sigma_{q}^{2}+\left(1-\alpha \right)\sigma_{p}^{2}]}\right\}\\ & ={\left(2\pi \sigma_{\alpha }^{2}\right)}^{\frac{\alpha +1-\alpha }{2}}{\left(2\pi \sigma_{p}^{2}\right)}^{-\frac{\alpha }{2}}{\left(2\pi \sigma_{q}^{2}\right)}^{-\frac{1-\alpha }{2}}exp\left\{-\frac{\alpha \left(1-\alpha \right){\left(\mu_{p}-\mu_{q}\right)}^{2}}{2[\alpha \sigma_{q}^{2}+\left(1-\alpha \right)\sigma_{p}^{2}]}\right\}\\ & ={\left(\frac{\sigma_{q}^{2}}{\alpha \sigma_{q}^{2}+\left(1-\alpha \right)\sigma_{p}^{2}}\right)}^{\frac{\alpha }{2}}{\left(\frac{\sigma_{p}^{2}}{\alpha \sigma_{q}^{2}+\left(1-\alpha \right)\sigma_{p}^{2}}\right)}^{\frac{1-\alpha }{2}}exp\left\{-\frac{\alpha \left(1-\alpha \right){\left(\mu_{p}-\mu_{q}\right)}^{2}}{2[\alpha \sigma_{q}^{2}+\left(1-\alpha \right)\sigma_{p}^{2}]}\right\} \end{array}$$

Therefore, $p_{\alpha }\left(x\right)$ is a normalized probability density function, satisfying the normalization conditions $\int p_{\alpha }\left(x\right)dx=1$. It is clear that the product of two Gaussian distributions is still a Gaussian distribution, which will bring great convenience to the representation of probability distribution of the latter filtering problem.

At the same time, we can get that the variance of $p_{\alpha }\left(x\right)$ is $\sigma_{\alpha }^{2}$, which should satisfy the condition that its value is greater than zero. We can know by its denominator when $\sigma_{q}^{2}\geq \sigma_{p}^{2}$, the value of α can take any value on the real number axis; when $\sigma_{q}^{2}<\sigma_{p}^{2}$, the scope of α is $\alpha <\frac{\sigma_{p}^{2}}{\sigma_{p}^{2}-\sigma_{q}^{2}}$. Then, it is easy to know that the closer $\sigma_{p}^{2}$ is to $\sigma_{q}^{2}$, the greater the range of values of α.

In addition, the influence of the mean and the variance of the two distributions on the mean and variance of the α-mixed probability density function can be analyzed to facilitate the solution of the algorithm latter. As for the variance, when $\sigma_{q}^{2}>\sigma_{p}^{2}$, $\sigma_{\alpha }^{2}$ decreases with the increase of α; when $\sigma_{q}^{2}=\sigma_{p}^{2}$, it can be concluded that $\sigma_{\alpha }^{2}=\sigma_{q}^{2}=\sigma_{p}^{2}$; when $\sigma_{q}^{2}<\sigma_{p}^{2}$, $\sigma_{\alpha }^{2}$ increases with the increase of α. As for the mean value, when $\sigma_{q}^{2}=\sigma_{p}^{2}$, $\mu_{\alpha }=\left(\mu_{p}-\mu_{q}\right)\alpha +\mu_{q}$; if $\sigma_{q}^{2}\neq \sigma_{p}^{2}$, $\mu_{\alpha }=\frac{\mu_{p}\sigma_{q}^{2}-\mu_{q}\sigma_{p}^{2}}{\sigma_{q}^{2}-\sigma_{p}^{2}}+\frac{\left(\mu_{q}-\mu_{p}\right)\sigma_{q}^{2}\sigma_{p}^{2}}{{\left(\sigma_{q}^{2}-\sigma_{p}^{2}\right)}^{2}\alpha +\left(\sigma_{q}^{2}-\sigma_{p}^{2}\right)\sigma_{p}^{2}}$. It is clear that if $\mu_{p}>\mu_{q}$, then $\mu_{\alpha }$ increases with the increase of α; if $\mu_{p}<\mu_{q}$, then $\mu_{\alpha }$ decreases with the increase of α. The summary of the properties is shown in Table 1.

**Figure 2:**
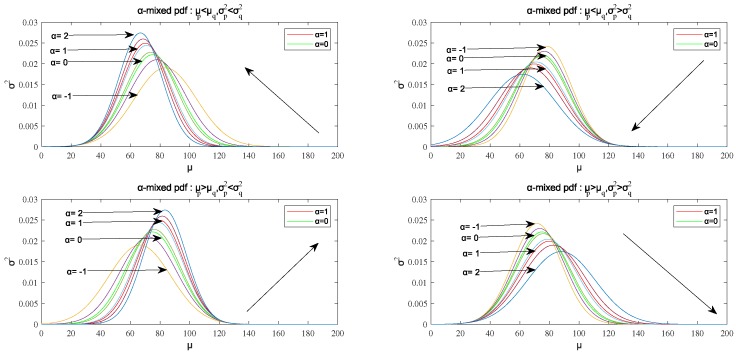
The monotonicity of the mean $\mu_{\alpha }$ and the variance $\sigma_{\alpha }^{2}$ with respect to α.

The monotonicity of the mean $\mu_{\alpha }$ and the variance $\sigma_{\alpha }^{2}$ with respect to α is shown in Figure 2.

It is clear that when $\mu_{p}<\mu_{q}$ and $\sigma_{q}^{2}>\sigma_{p}^{2}$, $\mu_{\alpha }$ decreases with the increase of α and $\sigma_{\alpha }^{2}$ decreases with the increase of α; when $\mu_{p}<\mu_{q}$ and $\sigma_{q}^{2}<\sigma_{p}^{2}$, $\mu_{\alpha }$ decreases with the increase of α and $\sigma_{\alpha }^{2}$ increases with the increase of α; when $\mu_{p}>\mu_{q}$ and $\sigma_{q}^{2}>\sigma_{p}^{2}$, $\mu_{\alpha }$ increases with the increase of α and $\sigma_{\alpha }^{2}$ decreases with the increase of α; when $\mu_{p}>\mu_{q}$ and $\sigma_{q}^{2}<\sigma_{p}^{2}$, $\mu_{\alpha }$ increases with the increase of α and $\sigma_{\alpha }^{2}$ increases with the increase of α.

When α∈(0,1), the α-mixed probability density function is the interpolation function of p(x) and q(x), so its mean value and the variance are all between p(x) and q(x), as shown in Figure 2, and its image curve is also between them.

The above analysis will be used in the algorithm implementation of the sufficient condition in the non-linear filtering algorithm.

### 4.2. The Alpha-Divergence Minimization

In the solving process of the alpha-divergence minimization, either the posterior distribution itself or the calculation of the maximized posterior distribution is complex, so the approximate distribution q(x) with good characterization ability is often used to approximate the true posterior distribution p(x). As a result, a higher degree achieves better approximation. Here, we restrict the approximate distribution q(x) to be an exponential family distribution; denote $p_{e}\left(x\right)$, with good properties, defined as follows:

(21) $$p_{e}\left(x\right)=h\left(x\right)exp\{\phi^{T}\left(\theta \right)u\left(x\right)+g\left(\phi \left(\theta \right)\right)\}$$

Here, θ is a parameter set of probability density function; c(x) and g(ϕ(θ)) are known functions; ϕ(θ) is a vector composed of natural parameters; u(x) is a natural statistical vector. u(x) contains enough information to express the state variable x in the exponential family distribution completely; ϕ(θ) is a coefficient parameter that combines u(x) based on parameter set θ.

In the non-linear filtering, assume the exponential family distribution is $p_{e}\left(x\right)$; arbitrary function is p(x), and we use $p_{e}\left(x\right)$ to approximate p(x), measuring the degree of approximation by the alpha-divergence. Therefore, the alpha-divergence of p(x) relative to $p_{e}\left(x\right)$ is obtained, defined as:

(22) $$\begin{array}{cc} J & =D_{\alpha }\left[p|\right|p_{e}]=\frac{1}{\alpha (1-\alpha )}\left[1-\int p{\left(x\right)}^{\alpha }p_{e}{\left(x\right)}^{1-\alpha }\right]\\ & =\frac{1}{\alpha (1-\alpha )}\left\{1-\int p{\left(x\right)}^{\alpha }{\left[h\left(x\right)exp\left(\phi^{T}\left(\theta \right)u\left(x\right)+g\left(\phi \left(\theta \right)\right)\right)\right]}^{1-\alpha }\right\} \end{array}$$

We state and prove in Theorem 1 that the alpha-divergence between the exponential family distribution and the probability density function of arbitrary state variable is minimum, if and only if the expected value of the natural statistical vector in the exponential family distribution is equal to the expected value of the natural statistical vector in the α-mixed probability state density function. In Corollary 1, given α=1, the equivalence condition can be obtained in the case of KL[p‖q]. In Corollary 2, we conclude that the specialization of the exponential family distribution is obtained after being processed by the Gaussian probability density function.

**Theorem** **1.** *The alpha-divergence between the exponential family distribution and the known state probability density function takes the minimum value; if and only if α≥1, the expected value of the natural statistical vector in the exponential family distribution is equal to the expected value of the natural statistical vector in the α-mixed probability state density function, that is:*

(23) $$E_{p_{e}}\left\{u(x)\right\}=E_{p_{\alpha }}\left\{u(x)\right\}$$

**Proof** **of** **Theorem** **1.** Sufficient conditions for J minimization are that the first derivative and the second derivative satisfy the following conditions:

(24) $$\frac{\partial J}{\partial \phi (\theta )}=0\;and\;\frac{\partial^{2}J}{\partial {\phi (\theta )}^{2}}>0$$

First, we derive Equation (22) with respect to ϕ(θ), and according to the conditions in the first derivative, the outcome is:

(25) $$\begin{array}{cc} \frac{\partial J}{\partial \phi (\theta )} & =\frac{-1}{\alpha (1-\alpha )}\int p{\left(x\right)}^{\alpha }\left(1-\alpha \right)p_{e}{\left(x\right)}^{-\alpha }p_{e}\left(x\right)\left\{u\left(x\right)+\left(\frac{\partial g(\phi (\theta ))}{\partial \phi (\theta )}\right)\right\}dx\\ & =\frac{-1}{\alpha }\int p{\left(x\right)}^{\alpha }p_{e}{\left(x\right)}^{1-\alpha }\left\{u\left(x\right)+\left(\frac{\partial g(\phi (\theta ))}{\partial \phi (\theta )}\right)\right\}dx\\ & =-\frac{1}{\alpha }\int p{\left(x\right)}^{\alpha }p_{e}{\left(x\right)}^{1-\alpha }u\left(x\right)dx-\frac{1}{\alpha }\int p{\left(x\right)}^{\alpha }p_{e}{\left(x\right)}^{1-\alpha }\left(\frac{\partial g(\phi (\theta ))}{\partial \phi (\theta )}\right)dx \end{array}$$

Let the above equation be equal to zero, then:

(26) $$\frac{\partial g(\phi (\theta ))}{\partial \phi (\theta )}=-\int \frac{p{\left(x\right)}^{\alpha }p_{e}{\left(x\right)}^{1-\alpha }}{\int p{\left(x\right)}^{\alpha }p_{e}{\left(x\right)}^{1-\alpha }dx}u\left(x\right)dx=-\int p_{\alpha }\left(x\right)u\left(x\right)dx$$

In addition, since $p_{e}\left(x\right)$ is a probability density function, it satisfies the normalization condition:

(27) $$\int p_{e}\left(x\right)dx=\int h\left(x\right)exp\{\phi^{T}\left(\theta \right)u\left(x\right)+g\left(\phi \left(\theta \right)\right)\}dx=1$$

Derive ϕ(θ) in the above equation, and the outcome is:

(28) $$\frac{\partial }{\partial \phi (\theta )}p_{e}\left(x\right)=\int p_{e}\left(x\right)u\left(x\right)dx+\frac{\partial g(\phi (\theta ))}{\partial \phi (\theta )}=0$$

The first item of Equation (23) can be obtained from Equations (26) and (28), which is the existence conditions of the stationary point for J.

To ensure that Equation (24) can minimize Equation (22), which means the stationary point is also its minimum point, we also need to prove that the second derivative satisfies the condition. Derive ϕ(θ) in Equation (25); the outcome is:

(29) $$\begin{array}{cc} \frac{\partial^{2}J}{\partial {\phi (\theta )}^{2}} & =\frac{\partial }{\partial \phi (\theta )}\left\{-\frac{1}{\alpha }\int p{\left(x\right)}^{\alpha }p_{e}{\left(x\right)}^{1-\alpha }u\left(x\right)dx-\frac{1}{\alpha }\int p{\left(x\right)}^{\alpha }p_{e}{\left(x\right)}^{1-\alpha }\left(\frac{\partial g(\phi (\theta ))}{\partial \phi (\theta )}\right)dx\right\}\\ & =-\frac{1-\alpha }{\alpha }\int p{\left(x\right)}^{\alpha }p_{e}{\left(x\right)}^{1-\alpha }\left\{u\left(x\right)+\left(\frac{\partial g(\phi (\theta ))}{\partial \phi (\theta )}\right)\right\}u\left(x\right)dx\\ & \;-\frac{1}{\alpha }\int p{\left(x\right)}^{\alpha }p_{e}{\left(x\right)}^{1-\alpha }\frac{\partial^{2}g\left(\phi \left(\theta \right)\right)}{\partial {\phi (\theta )}^{2}}dx\\ & \;-\frac{1-\alpha }{\alpha }\int p{\left(x\right)}^{\alpha }p_{e}{\left(x\right)}^{1-\alpha }\left\{u\left(x\right)+\left(\frac{\partial g(\phi (\theta ))}{\partial \phi (\theta )}\right)\right\}\frac{\partial g(\phi (\theta ))}{\partial \phi (\theta )}dx\\ & =-\frac{1}{\alpha }\int p{\left(x\right)}^{\alpha }p_{e}{\left(x\right)}^{1-\alpha }\frac{\partial^{2}g\left(\phi \left(\theta \right)\right)}{\partial {\phi (\theta )}^{2}}dx\\ & \;-\frac{1-\alpha }{\alpha }\int p{\left(x\right)}^{\alpha }p_{e}{\left(x\right)}^{1-\alpha }\left\{u{\left(x\right)}^{2}+2u\left(x\right)\left(\frac{\partial g(\phi (\theta ))}{\partial \phi (\theta )}\right)+{\left(\frac{\partial g(\phi (\theta ))}{\partial \phi (\theta )}\right)}^{2}\right\}dx\\ & =-\frac{1}{\alpha }\frac{\partial^{2}g\left(\phi \left(\theta \right)\right)}{\partial {\phi (\theta )}^{2}}\int p{\left(x\right)}^{\alpha }p_{e}{\left(x\right)}^{1-\alpha }dx+\frac{\alpha -1}{\alpha }\int p{\left(x\right)}^{\alpha }p_{e}{\left(x\right)}^{1-\alpha }{\left\{u\left(x\right)+\frac{\partial g(\phi (\theta ))}{\partial \phi (\theta )}\right\}}^{2}dx\\ & =-\frac{1}{\alpha }\frac{\partial^{2}g\left(\phi \left(\theta \right)\right)}{\partial {\phi (\theta )}^{2}}\int p_{\alpha }\left(x\right)dx+\frac{\alpha -1}{\alpha }\int p{\left(x\right)}^{\alpha }p_{e}{\left(x\right)}^{1-\alpha }{\left\{u\left(x\right)+\frac{\partial g(\phi (\theta ))}{\partial \phi (\theta )}\right\}}^{2}dx \end{array}$$

For the first item, it is easy to prove $\frac{\partial^{2}g\left(\phi \left(\theta \right)\right)}{\partial {\phi (\theta )}^{2}}<0$, and the proof is as follows.

It can be known from Equation (21):

(30) $$g\left(\phi \left(\theta \right)\right)=-log\int h\left(x\right)exp\left\{\phi^{T}\left(\theta \right)u\left(x\right)\right\}dx$$

The gradient of Equation (30) with respect to the natural parameter vector is as follows:

(31) $$\begin{array}{cc} \frac{\partial g(\phi (\theta ))}{\partial \phi (\theta )} & =-\int \frac{h\left(x\right)exp\left\{\phi^{T}\left(\theta \right)u\left(x\right)\right\}}{\int h\left(x\right)exp\left\{\phi^{T}\left(\theta \right)u\left(x\right)\right\}dx}u\left(x\right)dx=\\ & =-\int \frac{h\left(x\right)exp\left\{\phi^{T}\left(\theta \right)u\left(x\right)\right\}}{exp\left\{-g(\phi (\theta ))\right\}dx}u\left(x\right)dx=-\int p_{e}\left(x\right)u\left(x\right)dx \end{array}$$

Then, consider the matrix formed by its second derivative with respect to the natural parameter vector:

(32) $$\begin{array}{cc} \frac{\partial^{2}g\left(\phi \left(\theta \right)\right)}{\partial \phi^{i}\left(\theta \right)\partial \phi^{j}\left(\theta \right)} & =-\frac{\partial }{\partial \phi^{j}\left(\theta \right)}\int \frac{h\left(x\right)exp\left\{\phi^{T}\left(\theta \right)u\left(x\right)\right\}}{\int h\left(x\right)exp\left\{\phi^{T}\left(\theta \right)u\left(x\right)\right\}dx}u^{i}\left(x\right)dx\\ & =-\frac{\partial }{\partial \phi^{j}\left(\theta \right)}\frac{\int h\left(x\right)exp\left\{\phi^{T}\left(\theta \right)u\left(x\right)\right\}u^{i}\left(x\right)dx}{\int h\left(x\right)exp\left\{\phi^{T}\left(\theta \right)u\left(x\right)\right\}dx}\\ & =-\frac{\int h\left(x\right)exp\left\{\phi^{T}\left(\theta \right)u\left(x\right)\right\}u^{i}\left(x\right)dx\int h\left(x\right)exp\left\{\phi^{T}\left(\theta \right)\right\}u\left(x\right)dx}{{\left(\int h\left(x\right)exp\left\{\phi^{T}\left(\theta \right)u\left(x\right)\right\}dx\right)}^{2}}\\ & \;+\frac{\int h\left(x\right)exp\left\{\phi^{T}\left(\theta \right)u\left(x\right)\right\}u^{i}\left(x\right)dx\int h\left(x\right)exp\left\{\phi^{T}\left(\theta \right)u\left(x\right)\right\}u^{j}\left(x\right)dx}{{\left(\int h\left(x\right)exp\left\{\phi^{T}\left(\theta \right)u\left(x\right)\right\}dx\right)}^{2}}\\ & =-\left\{\int p_{e}\left(x\right)u^{i}\left(x\right)u^{j}\left(x\right)dx-\int p_{e}\left(x\right)u^{i}\left(x\right)dx\int p_{e}\left(x\right)u^{j}\left(x\right)dx\right\} \end{array}$$

According to the definition of the covariance matrix, the content in the bracket is the covariance matrix of the natural parameter vector with respect to the exponential family probability density function $p_{e}\left(x\right)$, and for arbitrary probability density distribution $p_{e}\left(x\right)$, the variance matrix is a positive definite matrix, so $\frac{\partial^{2}g\left(\phi \left(\theta \right)\right)}{\partial {\phi (\theta )}^{2}}$ < 0; and when α>0, the first item is greater than zero.

The integral of the second item is the secondary moment, so α≥1 or α<0, and the second item is greater than zero.

To sum up, when α≥1, $\frac{\partial^{2}J}{\partial {\phi (\theta )}^{2}}>0$. ☐

**Corollary** **1.** *(See Theorem 1 of [3] for more details) When α=1, $p_{\alpha }\left(x\right)=p\left(x\right)$, $D_{\alpha }\left[p\|q\right]$ turns into KL[p‖q]. We can obtain the above theorem under the condition of KL[p‖q] and obtain the approximate distribution by minimizing the KL divergence, which also proves that the stationary point obtained when the first derivative of its KL divergence is equal to zero also satisfies the condition that its second derivative is greater than zero. The corresponding expectation propagation algorithm is shown as follows:*

(33) $$E_{q(x)}\left\{u(x)\right\}=E_{p(x)}\left\{u(x)\right\}$$

**Corollary** **2.** *(See Corollary 1.1 of [3] for more details) When the exponential family distribution is simplified as the Gaussian probability density function, its sufficient statistic for $u\left(x\right)=\left(x,x^{2}\right)$, we use the mean and variance of Gaussian probability density function, and the expectation of the corresponding propagation algorithm can use the moment matching method to calculate, so the first moment and the second moment are defined as follows:*

(34) $$m=E_{p(x)}\left\{x\right\}\;and\;M=E_{p(x)}\left\{xx^{T}\right\}$$

*The corresponding second central moment is defined as follows:*

(35) $$P=M-mm^{T}=E_{p(x)}\left\{\left(x-m\right){\left(x-m\right)}^{T}\right\}$$

The complexity of Theorem 1 lies in that both sides of Equation (23) depend on the probability distribution of q(x) at the same time. The q(x) that satisfies the condition can be obtained by repeated iterative update on q(x). The specific process is shown in Algorithm 1:

**Algorithm 1** Approximation of the true probability distribution p(x).**Input:** Target distribution parameter of p(x); damping factor ϵ∈(0,1); divergence parameter α∈[1,+∞); initialization value of q(x) **Output:** The exponential family probability function q(x)1:Calculate the α-mixed probability density function $p_{\alpha }\left(x\right)$2:According to Equation (23), we get a new q(x) using the expectation propagation algorithm described in Corollary 1, and the new q(x) is denoted as $q^{\prime }\left(x\right)$3:Revalue the q(x) as(36)$$q\left(x\right)=\frac{q{\left(x\right)}^{\epsilon }q^{\prime }{\left(x\right)}^{1-\epsilon }}{\int q{\left(x\right)}^{\epsilon }q^{\prime }{\left(x\right)}^{1-\epsilon }dx}$$4:**while**KL[p‖q]>0.01**do**5:  Calculate the KL divergence of the old $q^{\prime }\left(x\right)$ and the new q(x)6:**end while**

In the above algorithms, we need to pay attention to the following two problems: giving an initial value of q(x) and selecting damping factors. As for the first problem, we can know that when $\sigma_{q}^{2}<\sigma_{p}^{2}$, the value range of α is $\alpha <\frac{\sigma_{p}^{2}}{\sigma_{p}^{2}-\sigma_{q}^{2}}$, according to the analysis of the α-mixed probability density function in Section 4.1. Although the value of α is greater than one, the value range of α is limited under the condition that $\sigma_{p}^{2}$ is unknown in the initial state; when $\sigma_{q}^{2}\geq \sigma_{p}^{2}$, the value of α can take any value on the whole real number axis, so the initial value we can choose is relatively larger, making $\sigma_{q}^{2}\geq \sigma_{p}^{2}$ and $\mu_{q}>\mu_{p}$. When the value of α is greater than one, the mean value of the α-mixed probability density function will decrease, and the variance will also decrease, as shown in the upper left of Figure 2.

As for the second question, when α∈(0,1), the α-mixed probability density function is the interpolation function of p(x) and q(x) according to the analysis in Section 4.1. The value range in (0,1) of damping factor ϵ is quite reasonable because the two probability density functions are interpolated when the value range of ϵ is in (0, 1), and the new probability density function is between the two. According to Equation (36), the smaller of ϵ, the closer the new q(x) to the old q(x); the larger of ϵ, the closer the new q(x) to $q^{\prime }\left(x\right)$. The mean value and the variance of $q^{\prime }\left(x\right)$ is smaller than the real p(x) according to the analysis of the first question. Then, we will continue to combine new q(x) with p(x) to form a α-mixed probability density function. Similarly, we clarify that the mean value and the variance of the new q(x) are larger than p(x), so the value of ϵ we choose should be as close as possible to one.

The convergence of the algorithm can be guaranteed after considering the above two problems, and we can get q(x) that meets the conditions. It can be known from Theorem 1 that the approximation q(x) of p(x) can be obtained to ensure it converges on this minimum point after repeated iterative updates.

### 4.3. Non-Linear Filtering Algorithm Based on the Alpha-Divergence

In the process of non-linear filtering, assuming that a priori and a posteriori probability density functions satisfy the Assumed Density Filter (ADF), then define the prior parameter as $\theta_{k}^{-}=\left\{m_{k}^{-},P_{k}^{-}\right\}$; the corresponding distribution is prior distribution $q(x_{k};\theta_{k}^{-})$; define the posterior parameter as $\theta_{k}^{+}=\left\{m_{k}^{+},P_{k}^{+}\right\}$, then the corresponding distribution is posterior distribution $q(x_{k};\theta_{k}^{+})$.

The prediction of the state variance can be expressed as follows:

(37a) $$p\left(x_{k}|z_{1:k-1}\right)=\int p\left(x_{k}|x_{k-1},z_{1:k-1}\right)dx_{k-1}$$

(37b) $$\theta_{k}^{-}=arg\min_{\theta }D_{\alpha }[p\left(x_{k}|z_{1:k-1}\right)\|q\left(x_{k};\theta \right)]$$

The corresponding first moment about the origin $f\left(x_{k-1}\right)=\int x_{k}p\left(x_{k}|z_{1:k-1}\right)dx_{k}$ of $p(x_{k}|z_{1:k-1})$ can be obtained from Equation (37a).

By Corollary 2, when the alpha-divergence is simplified to the KL divergence, the corresponding mean value and variance are:

(38a) $$m_{k}^{-}=\int f\left(x_{k-1}\right)q\left(x_{k-1};\theta_{k-1}^{+}\right)dx$$

(38b) $$P_{k}^{-}=\int f\left(x_{k-1}\right)f{\left(x_{k-1}\right)}^{T}N\left(x_{k-1}|m_{k-1}^{+},P_{k-1}^{+}\right)dx-m_{k}^{-}m_{k}^{-T}+Q_{k}$$

Here, the prior distribution $q(x_{k};\theta_{k}^{-})$ can be obtained.

Similarly, the update steps of the filter can be expressed as follows:

(39a) $$p\left(x_{k}|z_{1:k}\right)=\frac{p\left(z_{k}|x_{k},z_{1:k-1}\right)q\left(x_{k};\theta_{k}^{-}\right)}{\int p\left(x_{k}|x_{k},z_{1:k-1}\right)q\left(x_{k};\theta_{k}^{-}\right)dx_{k}}$$

(39b) $$\theta_{k}^{+}=arg\min_{\theta }D_{\alpha }[p\left(x_{k}|z_{1:k}\right)\|q\left(x_{k};\theta \right)]$$

It is clear according to Theorem 1:

(40) $$\begin{array}{cc} E_{q(x_{k};\theta_{k}^{+})}\left\{u(x)\right\} & =E_{p_{\alpha }\left(x\right)}\left\{u(x)\right\}=\int p_{\alpha }\left(x\right)u\left(x\right)dx=\int u\left(x\right)\frac{p{\left(x_{k}|z_{1:k}\right)}^{\alpha }q{\left(x_{k};\theta_{k}^{+}\right)}^{1-\alpha }}{\pi (x)}\pi \left(x\right)dx\\ & \approx \sum_{i=1}^{N}u\left(x^{i}\right)\frac{{\left[p\left(z_{k}|x_{k}^{i},z_{1:k-1}\right)q\left(x_{k}^{i};\theta_{k}^{-}\right)\right]}^{\alpha }q{\left(x_{k}^{i};\theta_{k}^{+}\right)}^{\left(1-\alpha \right)}/\pi \left(x^{i}\right)}{\sum_{j}{\left[p\left(z_{k}|x_{k}^{j},z_{1:k-1}\right)q\left(x_{k}^{j};\theta_{k}^{-}\right)\right]}^{\alpha }q{\left(x_{k}^{j};\theta_{k}^{+}\right)}^{\left(1-\alpha \right)}/\pi \left(x^{j}\right)} \end{array}$$

Here, $x^{i}∼iid\pi_{t}\left(x_{t}\right)$, i=1,⋯,N, $\pi_{t}$ is the proposal distribution. We choose the proposal distribution as a priori distribution $q(x_{k};\theta_{k}^{-})$. We define $w^{i}={\left[p\left(z_{k}|x_{k}^{i},z_{1:k-1}\right)q\left(x_{k}^{i};\theta_{k}^{-}\right)\right]}^{\alpha }q{\left(x_{k}^{i};\theta_{k}^{+}\right)}^{1-\alpha }/\pi \left(x^{i}\right)$, $W=\sum_{j}w^{j}$, so:

(41) $$E_{q(x_{k};\theta_{k}^{+})}\left\{u(x)\right\}\approx \frac{1}{W}\sum_{i=1}^{N}w^{i}u\left(x^{i}\right)$$

An approximate calculation of the mean value and the variance for $q(x_{k};\theta_{k}^{+})$ is conducted:

(42a) $$m_{k}^{+}=\frac{1}{W}\sum_{i=1}^{N}w^{i}x^{i}$$

(42b) $$P_{k}^{+}=\frac{1}{W}\sum_{i=1}^{N}w^{i}\left(x^{i}-m_{k}^{i}\right){\left(x^{i}-m_{k}^{i}\right)}^{T}$$

Since Equation (40) contains $q(x_{k};\theta_{k}^{+})$ on both sides of the equation, we must use Algorithm 1 to conduct the iterative calculation to get the satisfied posterior distribution $q(x_{k};\theta_{k}^{+})$.

If α=1, the above steps can be reduced to a simpler filtering algorithm, as shown in [3].

In this process, we do not use the integral operation of the denominator in Equation (39a), but use the Monte Carlo integral strategy proposed in [15], as shown in Equation (40). We cannot conduct resampling, which greatly reduces the calculation.

## 5. Simulations and Analysis

According to Theorem 1, when α≥1, the non-linear filtering method we proposed is feasible theoretically. In the simulation experiment, the algorithm is validated by taking different values when α≥1. We name our proposed method as AKF and compare it with the traditional non-linear filtering methods such as EKF and UKF.

We choose the Univariate Nonstationary Growth Model (UNGM) [22] to analyze the performance of the proposed method. The system state equation is:

(43) $$x\left(k\right)=0.5x\left(k-1\right)+\frac{2.5x(k-1)}{1+x^{2}\left(k-1\right)}+8cos\left(1.2\left(k-1\right)\right)+w\left(k\right)$$

The observation equation is:

(44) $$y\left(k\right)=\frac{x^{2}\left(k\right)}{20}+v\left(k\right)$$

The equation of state is a non-linear equation including the fractional relation, square relation and trigonometric function relation. w(k) is the process noise with the mean value of zero and the variance of Q. The relationship between the observed signal y(k) and state v(k) in the measurement equation is also non-linear. v(k) is the observation noise with the mean value of zero and the variance of R. Therefore, this system is a typical system with non-linear states and observations, and this model has become the basic model for verifying the non-linear filtering algorithm [22,23].

In the experiment, we set Q = 10, R = 1 and set the initial state as p(x(1))=N(x(1);0,1).

**Figure 3:**
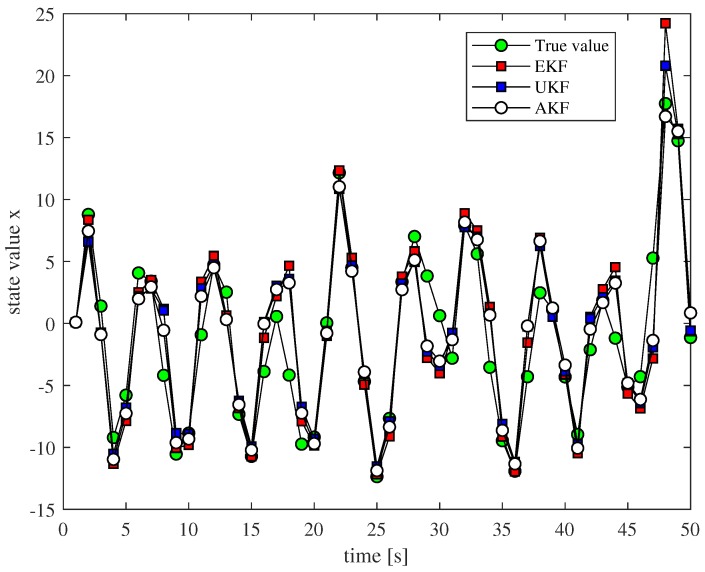
State estimation comparison of different non-linear filtering methods.

First, we simulate the system. When α≥1, the values of α are right for the experiments; here, the value of α is two, and the entire experimental simulation time is T = 50. The result of the state estimation is shown in Figure 3, and it can be seen that the non-linear filtering method we proposed is feasible; the state value can be estimated well during the whole process, and its performance is superior to EKF and UKF in some cases.

Second, in order to measure the accuracy of state estimation, the difference between the real state value at each moment and the estimated state value can be calculated to obtain the absolute value; thus, the absolute deviation of the state estimation at each moment is obtained, namely:

(45) $$RMS\left(k\right)=|x_{real}\left(k\right)-x_{estimated}\left(k\right)|$$

**Figure 4:**
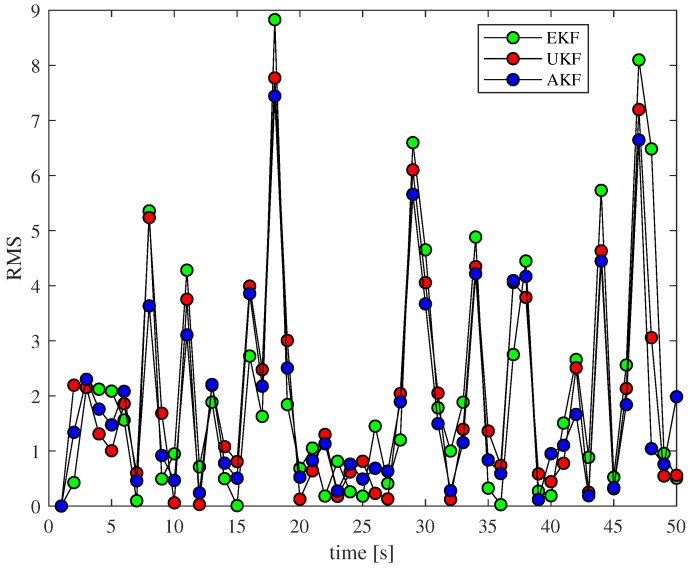
RMS comparison at different times.

As shown in Figure 4, we can see that the algorithm error we proposed is always relatively small where the absolute value deviation is relatively large. It can be seen that our proposed method performs better than other non-linear methods.

In order to measure the overall level of error, we have done many simulation experiments. The average error of each experiment is defined as:

(46) $$RMSE\left(k\right)=\frac{1}{T}\sum_{k-1}^{T}RMS\left(k\right)$$

The experimental results are shown in Table 2. We can see that when the estimation of T time series is averaged, the error mean of each AKF is minimum, which indicates the effectiveness of the algorithm, and the filtering accuracy of the algorithm is better than the other two methods under the same conditions. Because the UNGM has strong nonlinearity and we set the variance to the state noise as 10, which is quite large, so the performance differences between EKF, UKF and AKF are rather small.

Then, we analyze the influence of the initial value on the filtering results by modifying the value of process noise. As can be seen from Table 3, AKF’s performance becomes more and more similar to EKF/UKF as the Q becomes smaller.

**Figure 5:**
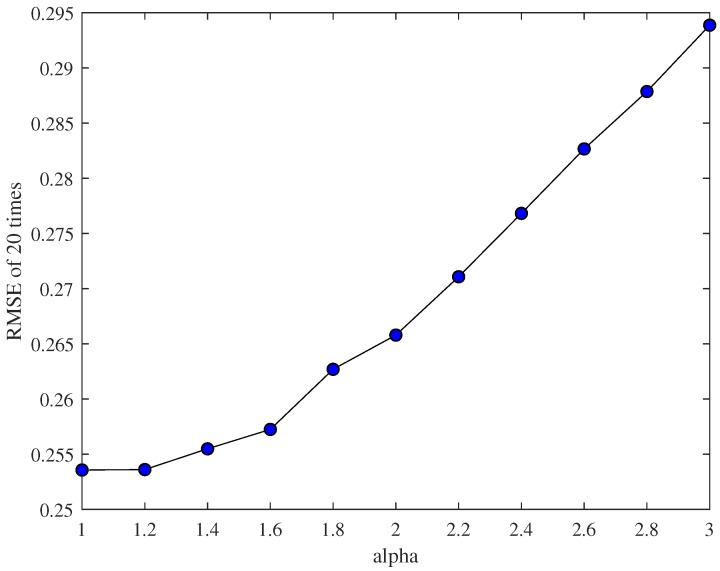
The error changes as α changes.

In the end, we analyze the performance of the whole non-linear filtering algorithm by adjusting the value of α through 20 experiments. In order to reduce the influence of the initial value on the experimental results, we take Q = 0.1 and then average the 20 experimental errors. The result is shown in Figure 5. We can see that the error grows as α grows in this example, as the noise is relatively small.

## 6. Conclusions

We have first defined the α-mixed probability density function and analyzed the monotonicity of the mean and the variance under different α values. Secondly, the sufficient conditions for α to find the minimum value have been proven, which provides more methods for measuring the distribution similarity of non-linear filtering. Finally, a non-linear filtering algorithm based on the alpha-divergence minimization has been proposed by applying the above two points to the non-linear filtering. Moreover, we have verified that the validity of the algorithm in one-dimensional UNGM.

Although the filtering algorithm is effective, the alpha-divergence is a direct extension of the KL divergence. We can try to verify that the minimum physical meaning of the alpha divergence is equivalent to the minimum physical meaning of the KL divergence in a further study. The algorithm should be applied to more practical applications to prove its effectiveness. Meanwhile, we can use more sophisticated particle filtering techniques, such as [24,25], to make the algorithm more efficient. Furthermore, the alpha-divergence method described above is applied to uni-modal approximations, but more attention should be paid to multi-modal distributions, which are more difficult and common in practical systems. Furthermore, it is worth designing a strategy to automatically learn the appropriate α values.

## Figures and Tables

**Table 1:** The monotonicity of the mean $\mu_{\alpha }$ and the variance $\sigma_{\alpha }^{2}$ of the α-mixed probability density function.

	$\sigma_{q}^{2}<\sigma_{p}^{2}$	$\sigma_{q}^{2}=\sigma_{p}^{2}$	$\sigma_{q}^{2}>\sigma_{p}^{2}$
	$\sigma_{\alpha }^{2}$ Increases with the Increase of α	$\sigma_{\alpha }^{2}=\sigma_{q}^{2}=\sigma_{p}^{2}$	$\sigma_{\alpha }^{2}$ Decreases with the Increase of α
$\mu_{p}>\mu_{q}$	$\mu_{\alpha }$ increases with the increase of α		
$\mu_{p}=\mu_{q}$	$\mu_{\alpha }=\mu_{p}=\mu_{q}$		
$\mu_{p}<\mu_{q}$	$\mu_{\alpha }$ decreases with the increase of α		

**Table 2:** Average errors of experiments.

	1	2	3	4	5	6	7
EKF	1.6414	1.8434	1.8245	1.7749	1.6666	1.3255	⋯
UKF	1.5400	1.7703	1.6688	1.6387	1.6241	1.2243	⋯
AKF	1.4819	1.5921	1.4710	1.4694	1.4389	1.1222	⋯

**Table 3:** Influence of the variance Q of state equation noise on experimental error.

Q	0.05	0.1	1	10
EKF	0.2256	0.2950	0.7288	1.7827
UKF	0.2222	0.3002	0.7396	1.6222
AKF	0.2167	0.2767	0.7144	1.5244
